# A Multifaceted Approach to Health Crisis in the Philippines: Comment on "Experiences and Implications of the First Wave of the COVID-19 Emergency in Italy: A Social Science Perspective"

**DOI:** 10.34172/ijhpm.8600

**Published:** 2024-08-19

**Authors:** Dalmacito A. Cordero Jr.

**Affiliations:** Department of Theology and Religious Education (DTRE), De La Salle University, Manila, Philippines.

**Keywords:** COVID-19, Epidemiological, Italy, Philippines, Religiosity, Social Science

## Abstract

The COVID-19 pandemic changed the landscape of every country’s healthcare system. May it be a developed nation like Italy or a developing one, like Philippines, it has seriously impacted the different aspects of people’s everyday living. While the epidemiological/pharmacological interventions is the top priority to battle the health crisis, considering the social science perspective in crafting any health policy is a must to develop holistic, relevant, and effective programs for policy-makers. This is not to underestimate the effectiveness of the former but it simply signifies the essential support of the latter for a higher success rate since it is a multifaceted approach. It is important to note that a human is not only composed of physicality, but all the other aspects that are connected to one another, including social, political, and spiritual ones.

 Masino and Enria recently published a paper about the first wave of the COVID-19 pandemic in Italy, focusing on its implications and experiences of the locals.^1^ The authors reflectively determined why it resulted in a devastating impact despite Italy’s classification as a “developed country.” They concluded that aside from epidemiological perspective, the socio-economic and political aspects must be considered for effective interventions. They enumerated several implications for policy-makers and the public, which will be the subject of my commentary. I aim to elaborate and enrich some of these claims for policy-makers not only from countries with robust healthcare systems but especially for those in constant struggle every time a health crisis takes place, like the Philippines.

 As a background, Italy was the first among European countries to experience the gruesome effect of COVID-19, with the first case declared on February 6, 2020. After two years, there had been more than 14 million confirmed cases and more than 156 000 deaths. This number is validated by the 229 000 compensation claims of healthcare workers and 835 deaths related to occupational exposure to the disease as of February 28, 2022.^2^ In the first implication to policy-makers, the authors pointed out that national epidemic preparedness plans needed to be moredequate concerning personnel and equipment supplies. Though the Italian government declared a national emergency on January 31, 2020, the national emergency plan requires a firm political will to impose strict lockdowns on time to control the virus’ spread. It has a low level of compliance, with those affected people from the northern regions traveling freely towards the south.^3^ This is why the estimated excess deaths in Lombardy reached more than 23 000 two months after the first wave. In the Philippines, the first confirmed COVID-19 case was reported on January 30, 2020. Strict lockdowns and travel restrictions were imposed two days earlier. However, travelers from the list of unrestricted countries were not required for screening and quarantine protocols. With this, the number of confirmed cases increased after a few weeks.^4^ It seemed like the Philippine government lacked the foresight of treating the disease as highly contagious, that the virus can easily spread to anyone and anywhere. This is where the role of political will plays an essential role. Political will refers to the decision-maker’s firm determination to cause change and attain desired results. Thus, it signifies the government’s utmost commitment to implement effectively its policies, especially in challenging situations.^5^ No matter how meaningful and appropriate the healthcare policies are, if the government lacks the will to implement them unconditionally, then it will have a lesser impact.^6^ This lack of political will was manifested in some of the government programs like the violent campaign on illegal drugs which destroys the well-being of every person. During the time of the previous administration, the ones who were commonly prosecuted and penalized are not the bigtime drug dealers but the poor and small-time pushers in the country. The government failed to enforce the full force of the law and did not have the will to run after the rich drug lords. If there is no political will in implementing any health program, the result will always be temporary and will not have a lasting effect.

 Another application of social science perspective that can be done by the government is establishing smaller communities that will have continuous connections every time there is an impending health crisis or even after a calamity. These communities can meet in-person to talk and share about their experiences and other inspiring stories about the challenging situation that they faced. This social interaction strategy can provide as a means of social support for one another in the midst of trying times. In fact, this can also be done virtually if the in-person set-up is not possible. With the availability of various online chat groups and social media platforms, this is always possible given the reality that majority of Filipinos are known to be one of the top social media users globally.

 Politically, the government can create more programs that are reward/incentive-based like organizing contest/competition to all families in a particular setting that will bring out their unique and creative ways of preventing a disease or promoting cleanliness in the household and within their vicinity. The reward can be in form of cash, grocery items, and even tax exemptions/cuts. This strategy will surely be “enticing” to the public, especially the poverty level in the country is still a serious problem.

 The authors also stressed the use of context-specific measures to increase local relevance and effectiveness in addressing health inequalities. They cited, for example, that social distancing and containment measures in some urban areas are challenging due to very high levels of population density and there is very limited open space for every family. I firmly support this claim and echo that health inequalities indeed exist in every country, and the way they are addressed or neglected is somehow a reflection of how robust or weak the healthcare system is. These disparities must be supported according to their context and needs. Those living with comorbidities or pre-existing medical conditions and with chronic diseases must be the priority of policy-makers, especially when it comes to vaccinations, rehabilitation, and treatment. Their vulnerable condition needs immediate attention to prevent the progression of their disease. Since they have a high risk of developing psychological distress and high levels of anxiety, follow-up mental health care is also needed.^7^ Those who belong to the poorest households and are under extreme financial constraints must be helped with emergency cash or even free food supply. This was inspiringly experienced in the Philippines during the pandemic, where government support was hardly felt, and concerned citizens were the ones who found ways to help. They organized community food pantries in different locations, offering everyone free food and basic necessities, especially the poor, needy, and unemployed.

 The authors also emphasized the importance of social cohesion in the implementation of national containment measures. They suggested that to increase adherence to government policies, there must be an open and constructive communication between the public, task forces, and the government. This excellent insight implies the importance of collaboration between the various institutions in our society, with the public acting not simply as a recipient of services but as a cooperating element that promotes solidarity for a successful healthcare system. Italy survived the pandemic, though it took some time because thousands of Italians found ways to support each other. Some locals showcased their skills and allotted time by entertaining those children stuck at home through virtual narration of fairy tales. Some chefs prepared meals for the hungry and homeless people. The Voluntary Emergency Brigades was founded by grassroots organizations, which coordinates the youth to bring groceries and medicines to those who are in quarantine, older people, and other vulnerable people in Milan.^8^ This show of solidarity inspires and motivates the different institutions to bond together and create programs for the benefit of the public.

 Lastly, as I reflect on the situation of Italy and the Philippines during the pandemic, it dawned on me how the practice of religiosity played an essential role in overcoming the crisis. In a study conducted about churches’ closure for more than two months in Italy, every religious celebration was shifted to online, such as the Pope’s blessing and the celebration of Mass. The authors claimed that indeed, more people derive comfort from religious activities during hard times that are characterized by uncertainty.^9^ A similar experience is shown by many Filipinos, who are known to be religious since the country is predominantly a Christian nation. Even amid the lockdown, Filipinos have found creative ways to express their religiosity by celebrating various devotions inside their homes. They prepared altars with crucifix, candles, and flowers and then celebrate the liturgy, following the live television Masses.^10^ The various social media platforms and live radio/TV broadcasting became the main avenues for religious practices during the pandemic.

 Considering the social science perspective in crafting any health policy is a must to develop holistic, relevant, and effective programs for policy-makers. This does not mean that epidemiological/pharmacological interventions are to be less-prioritized or replaced. The latter remains the primary antidote during a health crisis, but the success rate will be higher if the former’s contribution is always considered. It is important to note that a human is composed of physicality and all other aspects connected to one another, including psychosocial and spiritual ones.

## Ethical issues

 Not applicable.

## Competing interests

 Author declares that he has no competing interests.

## References

[1] Masino S, Enria L (2023). Experiences and implications of the first wave of the COVID-19 emergency in Italy: a social science perspective. Int J Health Policy Manag.

[2] Buresti G, Rondinone BM, Gagliardi D (2022). The impact of the first wave of the COVID-19 pandemic on healthcare workers: an Italian retrospective study. Int J Environ Res Public Health.

[3] Bosa I, Castelli A, Castelli M (2022). Response to COVID-19: was Italy (un)prepared?. Health Econ Policy Law.

[4] Amit AML, Pepito VCF, Dayrit MM (2021). Early response to COVID-19 in the Philippines. Western Pac Surveill Response J.

[5] Post LA, Raile AN, Raile ED (2010). Defining political will. Polit Policy.

[6] Cordero DA Jr (2024). The crucial role of political will in advancing primary health care. Korean J Fam Med.

[7] Stafford O, Berry A, Taylor LK (2021). Comorbidity and COVID-19: investigating the relationship between medical and psychological well-being. Ir J Psychol Med.

[8] Perrone A. ‘The Strength of Ordinary People.’ The Creative Ways Italians Are Supporting Each Other During Their Coronavirus Lockdown. Time; 2020. https://time.com/5816650/italy-solidarity-coronavirus/.

[9] Alfano V, Ercolano S, Vecchione G (2023). In COVID we trust: the impact of the pandemic on religiousness-evidence from Italian regions. J Relig Health.

[10] Corpuz JCG, Sarmiento PJD (2021). Going back to basics: experiencing Domus ecclesiae (House Church) in the celebration of the liturgy during COVID-19. Pract Theol.

